# Case Report: Complicated Meckel‘s diverticulum masquerading as gastroenteritis: a near-fatal presentation in a toddler

**DOI:** 10.3389/fped.2026.1740773

**Published:** 2026-06-09

**Authors:** Zhijiang Zhou, Ping Ling, Rong Tang, Ye Chen, Yi Lin, Xuexin Wang, Sha Wang

**Affiliations:** Department of Pediatric Emergency and Intensive Care Unit, Guiyang Maternal and Child Health Care Hospital, Guiyang, China

**Keywords:** Meckel's diverticulum, septic shock, pediatric, strangulated bowel, internal hernia

## Abstract

**Background:**

Meckel's diverticulum poses diagnostic difficulties due to its nonspecific symptoms, which often resemble benign conditions like gastroenteritis.

**Case presentation:**

A 2-year-11-month-old girl presented with abdominal pain and vomiting, initially diagnosed as acute gastroenteritis. Within 12 h, her condition deteriorated rapidly with fever, lethargy, and circulatory shock. Laboratory findings included marked leukocytosis (36.05 × 10⁹/L), severe metabolic acidosis (pH 7.32, HCO₃⁻ 5.2 mmol/L), and elevated lactate (3.3 mmol/L). Emergency abdominal CT showed small bowel obstruction with a mid-abdominal mass and significant ascites. Emergency laparotomy revealed a strangulated Meckel's diverticulum with ileal necrosis, requiring segmental bowel resection. The postoperative course was complicated by refractory septic shock, septic cardiomyopathy, and multiple organ dysfunction syndrome, necessitating advanced support including mechanical ventilation, continuous renal replacement therapy, and plasma exchange.

**Conclusion:**

This case highlights the potential for rapid clinical deterioration in children with surgically correctable abdominal pathologies that initially appear benign. Recognizing red flags-such as rapid systemic toxicity, disproportionate metabolic acidosis, and abdominal distension-is essential to prompt immediate surgical evaluation and prevent catastrophic outcomes.

## Introduction

Meckel's diverticulum (MD) is the most common congenital gastrointestinal anomaly in children (1). MD occurs in approximately 2% of the population, with a slight male predominance (1). Although most individuals remain asymptomatic, complications such as bleeding, intussusception, intestinal obstruction, and perforation can occur, typically presenting in the first few years of life (1, 2). MD's diverse clinical manifestations can often mimic common childhood illnesses such as acute gastroenteritis or appendicitis, resulting in delays in diagnosis that can lead to severe complications including bowel strangulation and septic shock, making awareness of this entity essential for emergency physicians and pediatricians (1, 2). We describe the case of a previously healthy toddler whose initially mild gastrointestinal symptoms progressed rapidly to septic shock due to a strangulated MD.

## Case presentation

### Patient information

A previously healthy 2-year-11-month-old girl was brought to medical attention with a 24-hour history of intermittent abdominal pain and recurrent non-bilious vomiting.

### Clinical findings and timeline

Initial physical examination revealed normal vital signs and a non-tender abdomen. Bedside abdominal ultrasound showed no abnormalities. She received a diagnosis of acute gastroenteritis and was discharged with recommendations for symptomatic management.

Twelve hours later, the child returned with dramatic clinical worsening. Examination disclosed fever (39.5 °C), profound lethargy, and evidence of circulatory failure including tachycardia (210 bpm), prolonged capillary refill (5 s), and abdominal distension with absent bowel sounds.

### Diagnostic focus and assessment

Laboratory evaluation demonstrated significant leukocytosis (36.05 × 10⁹/L), severe metabolic acidosis (pH 7.32, HCO₃⁻ 5.2 mmol/L, base excess −20.9), elevated lactate (3.3 mmol/L), and markedly elevated troponin T (109.4 pg/mL). Emergency abdominal CT revealed small bowel obstruction with dilated and tortuous small bowel loops and substantial ascites (Figure 1).

**Figure 1:**
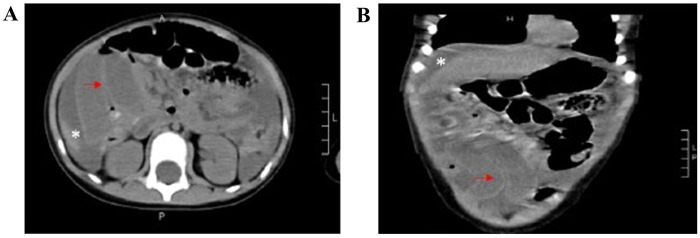
Preoperative abdominal CT imaging upon clinical deterioration. **(A)** Axial non-contrast CT image demonstrates dilated small bowel loops with prominent air-fluid levels (red arrow), confirming mechanical obstruction. Significant ascites is also present (white asterisk). **(B)** Coronal reformatted image shows dilated and tortuous small bowel loops (red arrow) proximal to the site of obstruction, with massive ascites (white asterisk).

Emergency laparotomy identified an internal hernia through a mesodiverticular band associated with a MD, causing ileal strangulation and necrosis (Figure 2). Segmental bowel resection with primary anastomosis was performed.

**Figure 2:**
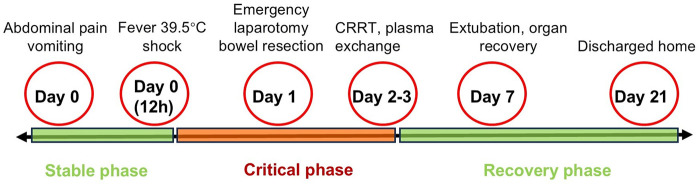
Timeline of clinical deterioration and multidisciplinary intervention. The patient initially presented with abdominal pain and vomiting and was discharged with a diagnosis of gastroenteritis. Within 12 h, she returned with fever (39.5 °C), shock, and abdominal distension. CT confirmed small bowel obstruction with ascites. Emergency laparotomy revealed a strangulated MD with ileal necrosis, requiring segmental bowel resection. Postoperatively, she developed septic shock, cardiomyopathy, and acute kidney injury, managed with mechanical ventilation, CRRT, and plasma exchange. She was discharged on day 21 with full recovery.

### Therapeutic focus and assessment

The patient developed refractory septic shock, septic cardiomyopathy (ejection fraction 44%), and acute kidney injury. Management required invasive mechanical ventilation, multiple vasoactive agents, and continuous renal replacement therapy. Plasma exchange was employed for coagulopathy management.

### Follow-up and outcomes

The patient's clinical status improved progressively, with successful discontinuation of organ support. The evolving patterns of key laboratory and functional parameters during hospitalization are summarized in Table 1. Follow-up imaging confirmed resolution of the obstruction and ascites. She was subsequently discharged in stable condition. The clinical course timeline is illustrated in Figure 3.

**Table 1:** Dynamic changes of key laboratory and functional parameters.

Parameter	Day 1 (Admission)	Day 1 (Post-op)	Day 3	Day 6	Reference range
Inflammation & Metabolism					
WBC ( × 10⁹/L)	36.05	11.95	7.67	11.81	5.0−17.0
Lactate (mmol/L)	3.3	3.8	2.1	1.1	< 2.0
pH	7.32	7.35	7.41	7.43	7.35–7.45
Myocardial Injury & Stress					
Ejection Fraction, EF (%)	44^a^	47	62	62	> 55
NT-proBNP (pg/mL)	770.8	8791.0	1607.0	32.70	< 125
CK-MB (U/L)	92.29	782.07	440.20	52.04	< 25
Troponin T (pg/mL)	109.4	387.3	27.20	13.43	< 14
Other Organ Functions					
ALT (U/L)	19	331	616	357	< 35
Creatinine (μmol/L)	65	76	13	15	20–50

a EF value obtained from initial bedside echocardiography. NT-proBNP, N-terminal pro-B-type natriuretic peptide; CK-MB, creatine kinase-mb isoenzyme. Laboratory values at day 1 (Post-op) represent the peak levels after surgery.

**Figure 3:**
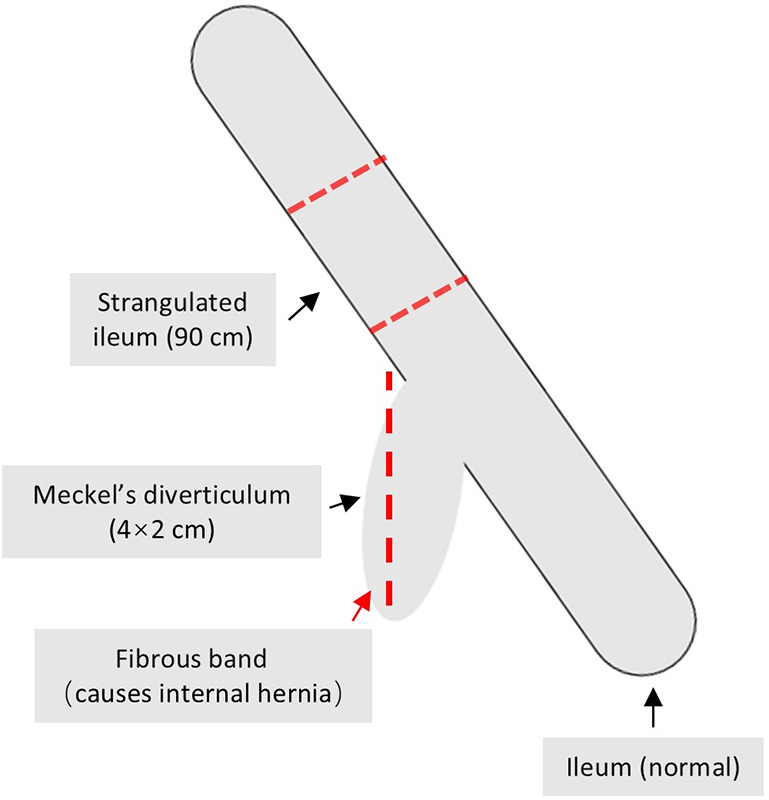
Intraoperative findings of strangulated MD. The image illustrates a MD (4 × 2 cm) with a fibrous band (red dashed line) that causes an internal hernia, leading to strangulation of approximately 90 cm of ileum (marked as “resected”). The adjacent normal ileum is also shown.

## Discussion

This case underscores important considerations in pediatric emergency care. The initial misdiagnosis of gastroenteritis represents a common clinical challenge, and the swift progression from benign presentation to septic shock within 12 h exemplifies the unpredictable nature of strangulated bowel obstruction (3). Several crucial indicators pointed toward surgical pathology: rapid emergence of systemic toxicity, metabolic acidosis disproportionate to the clinical picture, and new abdominal distension with absent bowel sounds. These clinical warning signs should trigger prompt surgical consultation (1–3).

This case also illustrates modern management approaches for severe sepsis with multi-organ dysfunction. Positive outcome relied on timely surgical intervention coupled with advanced critical care support, including continuous renal replacement therapy and plasma exchange (4, 5). Implementing these strategies promoted recovery from septic cardiomyopathy and multiple organ dysfunction.

From a diagnostic standpoint, this case highlights the limitations of bedside ultrasound for identifying surgical abdominal pathology, especially in early disease phases (6–9). While useful for excluding intussusception, ultrasound may overlook other causes of intestinal obstruction when performed early in the clinical progression. As McDonald et al. noted, CT imaging can provide more definitive diagnostic information in suspected complicated Meckel's diverticulum (9).

A review of the literature reveals that diagnostic delay is common in pediatric MD complicated by intestinal obstruction. Hansen and Søreide (1) reported that symptoms can be nonspecific, with approximately one-third of patients presenting with features mimicking acute abdomen of unclear etiology. Similar diagnostic challenges have been reported by Codrich and colleagues (10), who described a 7-year-old boy with a one-year history of recurrent abdominal pain and vomiting who was initially misdiagnosed with cyclic vomiting syndrome before intraoperative discovery of MD. Guanà and colleagues (11) also reported a 7-year-old girl with MD containing heterotopic pancreatic tissue who presented with intussusception and recurrent gastrointestinal bleeding, emphasizing the diagnostic difficulties even with advanced imaging. In our patient, the initial presentation of intermittent abdominal pain and vomiting without peritoneal signs was indistinguishable from viral gastroenteritis. The critical change occurred within 12 h when the child developed fever, tachycardia, and abdominal distension. These signs represent the transition from simple obstruction to strangulation with ischemia, which is a surgical emergency.

Several clinical red flags should alert clinicians to possible surgical pathology in a child presenting with vomiting. First, the rapid progression of symptoms within hours, as seen in this case, is highly unusual for uncomplicated gastroenteritis. Second, the presence of metabolic acidosis out of proportion to the degree of dehydration or fever suggests tissue ischemia. Third, the development of abdominal distension with absent bowel sounds indicates mechanical obstruction requiring imaging. When these features are present, immediate surgical consultation is warranted even if initial imaging appears inconclusive.

## Conclusion

This case reinforces the necessity of maintaining suspicion for surgical emergencies in children presenting with vomiting. MD, while uncommon, should remain in the differential diagnosis of abdominal pain in young children. Early identification of clinical warning signs and rapid multidisciplinary intervention are vital for optimal outcomes in these potentially devastating yet treatable conditions.

## Data Availability

The datasets presented in this study can be found in online repositories. The names of the repository/repositories and accession number(s) can be found in the article/Supplementary Material.
